# Dual-Cation Electrolytes Crosslinked with MXene for High-Performance Electrochromic Devices

**DOI:** 10.3390/nano11040874

**Published:** 2021-03-30

**Authors:** Soyoung Bae, Youngno Kim, Jeong Min Kim, Jung Hyun Kim

**Affiliations:** 1 Department of Chemical and Biomolecular Engineering, Yonsei University, 50 Yonsei-ro, Seodaemoon-Gu, Seoul 03722, Korea; sybae0922@yonsei.ac.kr (S.B.); jminjmin@yonsei.ac.kr (J.M.K.); 2 KIURI Institute, Yonsei University, 50 Yonsei-ro, Seodaemoon-Gu, Seoul 03722, Korea; dudsh3@naver.com

**Keywords:** MXene, poly(4-styrenesulfonic acid), Dual-Cation, polymer electrolyte, electrochromic

## Abstract

MXene, a 2D material, is used as a filler to manufacture polymer electrolytes with high ionic conductivity because of its unique sheet shape, large specific surface area and high aspect ratio. Because MXene has numerous -OH groups on its surface, it can cause dehydration and condensation reactions with poly(4-styrenesulfonic acid) (PSSA) and consequently create pathways for the conduction of cations. The movement of Grotthuss-type hydrogen ions along the cation-conduction pathway is promoted and a high ionic conductivity can be obtained. In addition, when electrolytes composed of a conventional acid or metal salt alone is applied to an electrochromic device (ECD), it does not bring out fast response time, high coloration efficiency and transmittance contrast simultaneously. Therefore, dual-cation electrolytes are designed for high-performance ECDs. Bis(trifluoromethylsulfonyl)amine lithium salt (LiTFSI) was used as a source of lithium ions and PSSA crosslinked with MXene was used as a source of protons. Dual-Cation electrolytes crosslinked with MXene was applied to an indium tin oxide-free, all-solution-processable ECD. The effect of applying the electrolyte to the device was verified in terms of response time, coloration efficiency and transmittance contrast. The ECD with a size of 5 × 5 cm^2^ showed a high transmittance contrast of 66.7%, fast response time (8 s/15 s) and high coloration efficiency of 340.6 cm^2^/C.

## 1. Introduction

Electrolytes serves as an ion conductor and separator for the electrode. These electrolytes are applied to various fields such as fuel cells, lithium cells and solar cells, etc. [1,2,3,4,5,6,7,8,9]. Among them, when the electrolyte is applied to an electrochromic device, a special transparent characteristic is required. Typical electrolytes used in electrochromic devices (ECDs) include liquid electrolytes and polymer electrolytes [10,11,12,13,14,15]. Liquid electrolyte is a representative obstacle in fabricating flexible devices and when exposed to mechanical strain, the electrolyte leaks out. On the other hand, the polymer electrolyte has the advantage of being resistant to mechanical strain. In addition, there is no need to seal the device. Electrolyte can be printed on the electrode through the solution process [16]. A polymer electrolyte is formed when a metal salt or acid is added to a polymer host and organic solvent [17,18]. To increase the ionic conductivity of the polymer electrolyte, numerous inorganic materials are introduced into the electrolyte as a filler [19,20,21]. The filler acts as a plasticizer to lower the crystallinity of the polymer and increases the movement of the polymer segment [22]. Fillers with abundant chemical functional groups dissociate salts through Lewis acid-base interaction and promote the movement of cations between fillers and polymer matrix [23]. In this study, MXene were used as a filler. MXene is produced by selectively etching the MAX phase, where M represents a transition metal, A is a group 13 or 14 element and X is C or N. MXene is terminated with -OH, -O- and -F. MXene, a 2D material, has a unique sheet shape, a large specific surface area and a high aspect ratio [24]; it is thus used as a filler to prepare a polymer electrolyte with high ionic conductivity. In this study, among the various types of MXene, Ti_3_C_2_T_X_ (T represents surfaces termination such as -O-, -OH and -F) was used. As MXene has a large number of -OH groups on its surface, it can cause dehydration and condensation reactions with poly(4-styrenesulfonic acid) (PSSA) and consequently create a pathway for the conduction of cations. The migration of Grotthuss-type hydrogen ions along the cation-conduction pathway can occur [24]. Therefore, electrolytes with higher ionic conductivity can be obtained.

In general, electrolytes that use metal salts or acids alone are not suitable for application in conductive polymer-based ECDs. Because Li^+^ cations form an electrical double-layer well, the diffusion rate is slow; therefore, the response time of the ECD is slow. H^+^ cations have a high diffusion rate because of their small size; therefore, the response time of the device is fast, but the electrical double layer cannot be well formed, decreasing the transmittance contrast. Therefore, an electrolyte was designed to obtain both of these advantages by simultaneously using a metal salt and an acid. Bis(trifluoromethylsulfonyl)amine lithium salt (LiTFSI) was used as a source of lithium ions and PSSA was used as a source of protons. The performance of the ECD using dual-cation electrolytes crosslinked with MXene was evaluated in terms of response time, coloration efficiency and transmittance contrast. The dual-cation electrolyte crosslinked with MXene was found to be a promising electrolyte platform for fabricating flexible ECDs, especially conductive polymer-based ECDs.

ECDs can change optical properties, such as transmittance, by applying a voltage. Common ECDs include anti-glare mirrors [25,26], smart glasses [27,28] and displays [29,30]. These have recently been applied in new applications such as electronic skins [31,32] and electrochromic supercapacitors [33,34]. When indium tin oxide (ITO), which is generally used, is employed as a transparent electrode, it is difficult to implement a flexible device. Therefore, conductive polymers are preferred over inorganic materials because of their adhesion to the substrate, flexibility and processability. The most widely studied conductive polymer is poly(3,4-ethylenedioxy thiophene): polystyrene sulfonate (PEDOT:PSS), which has excellent electrical properties and is an electrochromic material. Conventional ECDs consist of two electrodes: a cathodic species, an electrolyte and a secondary electrochromic (EC) film (as the anodic species). By using PEDOT:PSS as an electrode, the electrode and EC layer can be combined into one [35]. Poly(aniline): polystyrene sulfonate (PANI:PSS) can be used as a secondary EC film and ion storage layer. By directly adding benzyl viologen dichloride, an electrochromic material and 4-hydroxy-TEMPO, an anodic species, to the electrolyte, the number of layers is reduced and the stability and transmittance contrast of ECDs are improved.

## 2. Materials and Methods

### 2.1. Materials

Acrylamide (purity ≥98.0%), dimethyl sulfoxide (DMSO, ≥99.7%), poly(ethylene glycol) diacrylate (PEGDA, M_n_ = 700), 4-hydroxy-TEMPO (97%), benzyl viologen dichloride (97%), LiTFSI (99.95% trace metals basis) and 1-Hydroxycyclohexyl phenyl ketone (99%) were purchased from Sigma-Aldrich (Seoul, Korea). Poly(sodium 4-styrene sulfonate) (PSSNa, M_w_ ~ 1,000,000, 20 wt% in H_2_O) solution was purchased from Tosoh Corporation (Tokyo, Japan). A PSSA solution was prepared by treating PSSNa with an ion-exchange resin (TRILITE UPRC100U, Samyang Corporation). MXene (Ti_3_C_2_T_X_, 3–5µm) was purchased from Invisible Corporation (Suwon, Korea). MXene was made by etching Ti_3_AlC_2_ powder with 40% HF solution for 72 h.

### 2.2. Preparation of Electrolytes

#### 2.2.1. LiTFSI-Based Electrolytes (MXene/LiTFSI)

A homogeneous electrolyte solution was prepared as follows. A mixture of 2 g acrylamide, 2 g DMSO, 0.197 g PEGDA, 0.004 g 4-hydroxy-TEMPO, 0.028 g 1-hydroxycyclohexyl phenyl ketone, 0.1 g benzyl viologen dichloride, 0.011 g MXene and 0.2 g LiTFSI solution was prepared. The mixture was stirred for 30 min. A transparent polymer electrolyte solution was obtained. The optimized content of LiTFSI was used when the bleaching time of ECD was the fastest (Table S3). 

#### 2.2.2. Preparation of PSSA-Based Electrolytes Crosslinked with MXene (M-PSSA)

A homogeneous electrolyte solution was prepared as follows. A mixture of 2 g acrylamide, 2 g DMSO, 0.197 g PEGDA, 0.004 g 4-hydroxy-TEMPO, 0.028 g 1-hydroxycyclohexyl phenyl ketone, 0.1 g benzyl viologen dichloride, 0.011 g MXene and 4.3 g PSSA solution was prepared. The mixture was stirred for 30 min. A transparent polymer electrolyte solution was obtained. The content of PSSA was optimized to the maximum amount capable of maintaining the solid phase after UV curing for high ionic conductivity.

#### 2.2.3. Preparation of Dual-Cation Electrolytes Crosslinked with MXene (M-PSSA/LiTFSI)

A homogeneous electrolyte solution was prepared as follows. A mixture of 2 g acrylamide, 2 g DMSO, 0.197 g PEGDA, 0.004 g 4-hydroxy-TEMPO, 0.028 g 1-hydroxycyclohexyl phenyl ketone, 0.1 g benzyl viologen dichloride, 0.215 g LiTFSI, 0.011 g MXene and 4.3 g PSSA solution was prepared. The mixture was stirred for 24 h at 150 °C. A transparent polymer electrolyte solution was obtained. The optimal LiTFSI:PSSA ratio was determined in consideration of the transmittance contrast and response time (Table S4). 

### 2.3. Preparation of Electrochromic Electrodes

#### 2.3.1. Synthesis of PEDOT:PSS

The polymerization via in-situ PEDOT:PSS was performed, which was 3 eq. molar ratio of the EDOT monomer. After the sulfuric acid and poly(sodium-4-styrenesulfonate) (PSS) were dissolved in DI-water, the remainder of the EDOT, Fe_2_(SO_4_)_3_ and Na_2_S_2_O_8_ were injected into the reactor. The reaction temperature was 10 °C and the conditions were kept constant for 24 h under an argon atmosphere. The ratio of PEDOT to PSS was 1:2.5 wt%. After the reaction time of 24 h, cation and anion exchange resin was added to the reactor to remove the residual initiator and any other impurities of the reactions, such as sodium salt and stirred for 2 h. The solid contents of the acquired solution were adjusted to 1.0 wt%. After homogenization of the solution, a dark blue liquid solution was obtained in-situ PEDOT:PSS [36,37].

#### 2.3.2. Synthesis of PANI:PSS

The polymerization via H_2_SO_4_ in-situ PANI:PSS was performed using sulfuric acid, which was 1 eq. molar ratio of the Aniline monomer. After the sulfuric acid and poly(sodium-4-styrenesulfonate) (PSS) were dissolved in DI-water, the remainder of the Aniline, Fe_2_(SO_4_)_3_ and Na_2_S_2_O_8_ were injected into the reactor. The reaction temperature was 10 °C and the conditions were kept constant for 24 h under an argon atmosphere. The synthesis followed the Baytron P procedure and the ratio of Polyaniline to PSS was 1:2.5 wt%. After the reaction time of 24 h, cation and anion exchange resin was added to the reactor to remove the residual initiator and any other impurities of the reactions, such as sodium salt and stirred for 1.5 h. The solid contents of the acquired solution were adjusted to 1.5 wt%. After homogenization of the solution, a dark blue liquid solution was obtained: H_2_SO_4_ in-situ PANI:PSS [38].

#### 2.3.3. Fabrication of Electrochromic Electrodes

First, 5.0 wt% DMSO and 0.1 wt% surfactant were added to the PEDOT:PSS and PANI:PSS aqueous dispersions, stirred for 10 min and filtered through a 0.45 µm polypropylene syringe filter. This PEDOT:PSS dispersion was coated on a polyethylene terephthalate (PET) substrate using an RDS (R.D. Specialties) coating bar #15 (wet thickness: 34 µm) and dried in a convection oven at 150 °C for 4 min. The sheet resistance of the formed film was controlled to be approximately 35 Ω and it was used as the working electrode. The PEDOT:PSS dispersion was coated on a PET substrate using an RDS coating bar #4 (wet thickness: 9 µm) and dried in a convection oven at 150 °C for 4 min; thereafter, PANI:PSS dispersion was coated on the PEDOT:PSS using an RDS coating bar #11 (wet thickness: 25 µm). The sheet resistance of the formed film was controlled to be approximately 100 Ω, forming the counter electrode.

### 2.4. Fabrication of Flexible ECDs

PET films coated with PEDOT:PSS with an RDS #15 bar (wet thickness: 34 µm) were used as the working electrode and PET films coated with PEDOT:PSS with an RDS #4 bar (wet thickness: 9 µm) and PANI:PSS with an RDS #11 bar (wet thickness: 25 µm) were used as the counter electrode. The electrolyte solution was coated onto the working electrode using a doctor blade (wet thickness: 300 µm) and irradiated with ultra-violet (UV) light for 10 s. Then, the counter electrode was laminated on the polymer electrolyte to form the ECDs^35^.

### 2.5. Sample Characterization

To investigate the EC performance of the resulting devices, DC and square-wave voltages were applied using a potentiostat (AMETEK, PARSTAT MC, PMC1000). The ionic conductivity of electrolytes was measured by electrochemical impedance spectroscopy (EIS) in a stainless steel/electrolytes/stainless steel configuration. The areal capacitance was measured by galvanostatic charge discharge (GCD) measurements using a potentiostat (AMETEK, PARSTAT MC, PMC1000, Princeton, NJ, USA). UV-visible (UV-vis) spectra (200–1100 nm at a scan rate of 400 nm/min) were recorded at various applied voltages. The transmittance of the film and the device were measured using UV-vis-near-infrared (NIR) spectrophotometry (JASCO Corporation, V-650, Mary’s Court Easton, MD, USA) (AVANTES, Avalight-DHS). All measurements were performed under ambient conditions.

## 3. Results and Discussion

### 3.1. Synthesis and Characterization of PSSA Crosslinked with MXene

**Figure 1:**
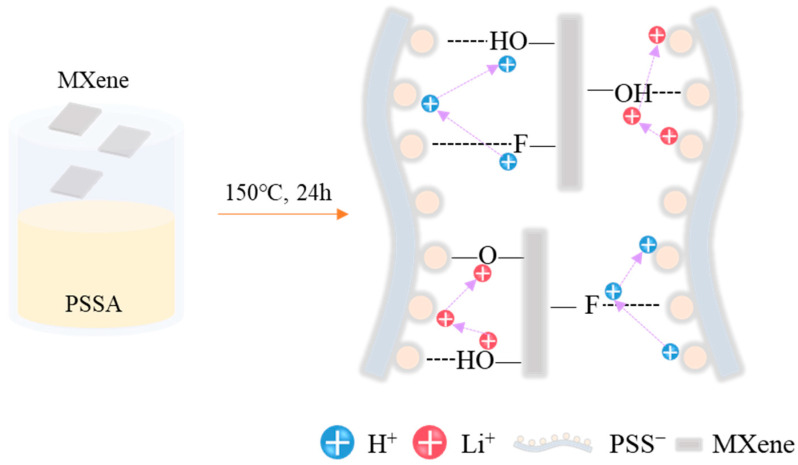
Schematic illustration of the fabrication of the poly(4-styrenesulfonic acid) crosslinked with MXene solution and proton conduction mechanism.

**Figure 2:**
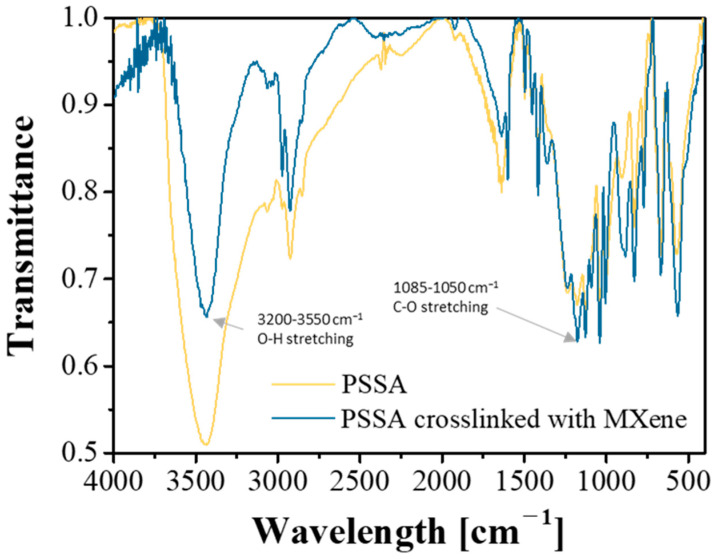
Fourier-Transform infrared spectroscopy spectra of the poly(4-styrenesulfonic acid) (PSSA) and PSSA crosslinked with MXene solution.

PSSA crosslinked with MXene (Ti_3_C_2_T_x_) was synthesized by mixing PSSA and MXene with sulfuric acid as the catalyst, followed by heating at 150 °C for 24 h (Figure 1). Unlike other 2D materials, such as graphene, the high aspect ratio of MXene, a large surface area and a surface with -OH, -F and -O- functional groups provides special properties when used as a filler. Owing to its hydrophilic surface properties, MXene is well dispersed in PSSA. In addition, by combining MXene and PSSA, the Grotthuss-type movement of cations, where cations hop from one carrier site to another, is promoted. This is because the cation transport path created between MXene and PSSA reduces the distance over which cations have to hop and creates a wide range of cation transport paths, greatly promoting the movement of cations inside the electrolyte. Fourier-transform infrared spectroscopy (FT-IR) analysis was used to confirm whether MXene and PSSA were crosslinked. According to Figure 2, the decrease in the peak intensity (OH stretching) in the 3200–3550 cm^−1^ region and the increase in the peak (CO stretching) intensity in the 1085–1050 cm^−1^ region resulted in the dehydration of the hydroxy group of MXene and the hydroxy group of PSSA [39,40]. The crosslinking was formed through a condensation reaction, showing that a chemical bond was formed. The ionic conductivity of the electrolyte according to the MXene content is as shown in Table S1 and the highest ionic conductivity was obtained when 0.25 wt% of MXene was added to the mass of PSSA. The measured capacitance of the device was highest when the MXene content was 0.25 wt%, similar to the trend in the ionic conductivity (Figure S1, Table S2).

### 3.2. Comparison of Electrochromic Performances in Lithium, Acid and Dual-Cation Electrolytes

When an electrolyte composed of a conventional acid or metal salt alone is applied to an ECD, it does not bring out fast response time, high coloration efficiency and transmittance contrast simultaneously [41]. Because Li^+^ cations form an electrical double-layer well, the diffusion rate is slow; therefore, the response time of the ECD is slow. H^+^ cations have a high diffusion rate because of their small size; therefore, the response time of the device is fast, but the electrical double layer cannot be well formed, decreasing the transmittance contrast. Therefore, an electrolyte was designed to obtain both advantages by simultaneously using a metal salt and an acid. LiTFSI was used as a source of lithium ions and PSSA crosslinked with MXene was used as a source of protons. Therefore, a dual-cation electrolyte crosslinked with MXene (M-PSSA/LiTFSI) is designed for high-performance ECDs. The electrolyte was applied to an ITO-free, all-solution-processable electrochromic device. The effect of applying the electrolyte to the device was verified in terms of response time, coloration efficiency and transmittance contrast.

**Figure 3:**
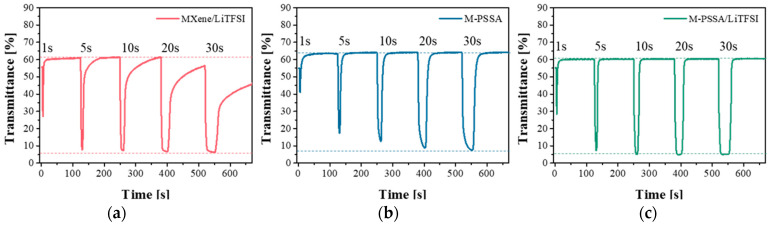
Transmittance vs. time of chronoamperometry at the wavelength of 600 nm of (**a**) MXene/Bis(trifluoromethylsulfonyl)amine lithium salt (MXene/LiTFSI), (**b**) poly(4-styrenesulfonic acid) crosslinked with MXene (M-PSSA) and (**c**) 1:20 LiTFSI in MXene-PSSA(M-PSSA/LiTFSI). Applied square-wave potential between −1.2 V (1, 5, 10, 20 and 30 s) and 0.5 V (120 s).

Figure 3 shows a measurement of the change in the transmittance of an ECD when a steady voltage is applied for a specific time. As shown in Figure 3a, the LiTFSI-based electrolyte (MXene/LiTFSI) quickly reached a complete coloration state even when a voltage of 1.2 V was applied for 5 s, but the original transmittance did not recover, even with a bleaching time of 120 s. According to Figure 3b, when the PSSA-based electrolyte(M-PSSA) was used, it had the advantage of being rapidly colored and bleached, but it had the disadvantage of only reaching a complete coloration state when a voltage of −1.2 V was applied for 30 s. When LiTFSI and M-PSSA were used together, the two advantages of fast coloration and bleaching could be obtained simultaneously, as shown in Figure 3c.

**Figure 4:**
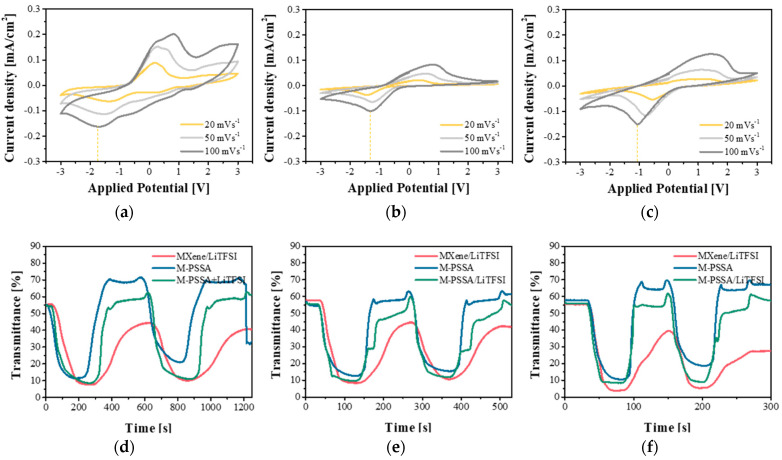
Cyclic voltammetry of (**a**) MXene/Bis(trifluoromethylsulfonyl)amine lithium salt (MXene/LiTFSI), (**b**) poly(4-styrenesulfonic acid) crosslinked with MXene (M-PSSA) and (**c**) 1:20 LiTFSI in MXene-PSSA(M-PSSA/LiTFSI) at different scan rates, and the corresponding transmittance vs time at the wavelengths of 600 nm for (**d**) 20 mV s^−1^, (**e**) 50 mV s^−1^, and (**f**) 100 mV s^−1^.

Changes in the CV curve and transmittance of the ECD at various scan rates and types of electrolytes were measured for comparing the response time of three electrolytes. In the case of a device with a fast response time, it will quickly reach a colored state and bleached state at any scan rate of 20–100 mV s^−1^. As the scan rate increased from 20 to 100 mV s^−1^, the current density increased equally for the three electrolytes (Figure 4a–c). However, the bleaching rate was different for the three electrolytes. In the case of MXene/LiTFSI (Figure 4d), it is difficult to desorb lithium ions inserted in the EC layer because it does not return to the initial transmittance during the bleaching process, regardless of the scan rate. It can be seen that the response time is slow. In contrast, in the case of M-PSSA (Figure 4e), it can be seen that it has a fast response time. However, it did not reach a completely colored state compared to the other electrolytes at a high scan rate. The M-PSSA/LiTFSI exhibited excellent coloration and bleaching characteristics regardless of the scan rate using LiTFSI and PSSA at a fast scan rate. In addition, the reduction peak appeared at a lower voltage, indicating that the coloration of the device began at a lower voltage.

**Figure 5:**
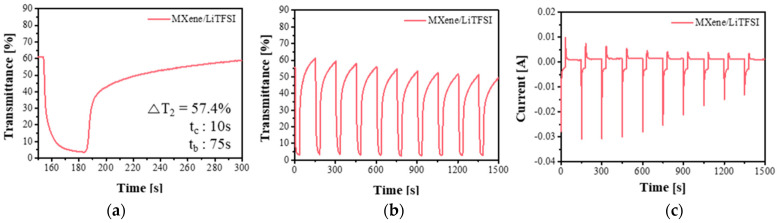

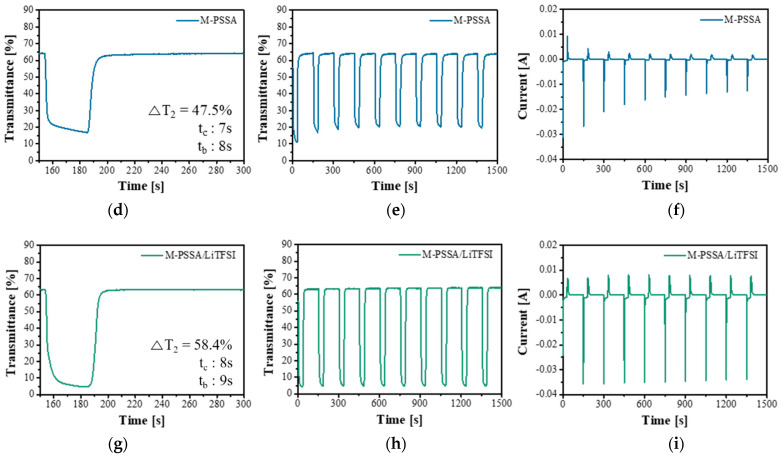
Transmittance vs. time of electrochromic devices (ECDs) of various types of electrolytes (**a**,**b**) MXene/Bis(trifluoromethylsulfonyl)amine lithium salt (MXene/LiTFSI), (**d**,**e**) poly(4-styrenesulfonic acid) crosslinked with MXene (M-PSSA) and (**g**,**h**) 1:20 LiTFSI in MXene-PSSA(M-PSSA/LiTFSI) and current vs. time of ECDs of various types of electrolytes (**c**) LiTFSI, (**f**) PSSA (**i**) 1:20 LiTFSI in PSSA at the wavelengths of 600 nm for square-wave potentials between −1.2 V (30 s) and 0.5 V (120 s).

A voltage of −1.2 V was applied for 30 s to the ECDs using three types of electrolytes to color the device and 0.5 V was applied for 120 s to bleach the device. In the case of MXene/LiTFSI (Figure 5a–c), the time required for 90% colorization was 10 s, the time required for a 90% bleached state was 75 s and the current value over time was not maintained under a constant condition, even after 10 cycles of operation and showed an unstable tendency. In the case of M-PSSA (Figure 5d–f), the bleaching speed was excellent because H^+^ is easily inserted and desorbed into working electrode. However, it had the disadvantage of not reaching a completely colored state when a voltage of −1.2 V was applied for 30 s. The ability of H^+^ to form a double layer is insufficient compared to LiTFSI. In addition, it can be seen that the current value gradually decreased with time over the 10 cycles operation. When LiTFSI and MXene-PSSA were used together (Figure 4g–i), both the coloration and bleaching speed were excellent and it can also be confirmed that the current value was stably maintained over 10 cycles of operation. LiTFSI provides a high ΔT and PSSA provides a fast response time. This tendency remained when ITO was used as a transparent electrode (Figure S2).

**Figure 6:**
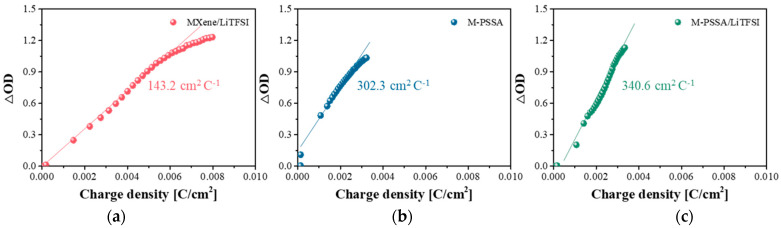
Coloration efficiency at the wavelengths of 600 nm of (**a**) MXene/Bis(trifluoromethylsulfonyl)amine lithium salt (MXene/LiTFSI), (**b**) poly(4-styrenesulfonic acid) crosslinked with MXene (M-PSSA) and (**c**) 1:20 LiTFSI in MXene-PSSA(M-PSSA/LiTFSI).

Figure 6 shows the data obtained by measuring the coloration efficiency of an ECD according to the three types of electrolytes. The coloration efficiency (CE) of the ECD can be obtained from the slope of the linear region [42]. CE is defined as ∆*OD*/∆*Q*, ∆*OD* = log(*T_b_*/*T_c_*), where ∆*OD* is the amount of change in optical density and ∆*Q* is the amount of inserted charge. *T_b_* and *T_c_* refer to the transmittance when colored and when bleached. The CE was largest when LiTFSI and MXene-PSSA were used together. This means that a large Δ*T* can be obtained even with a small amount of charge density.

In the open circuit, the change in transmittance over time was examined (Figure S3). This phenomenon was confirmed using Steps 1–4. In Zone 1, 0.5 V was applied until a completely bleached state was reached (maximum transmittance). In Zone 2, no voltage was applied until the initial transmittance was restored. In Zone 3, −1.2 V was applied until a completely colored state was reached (minimum transmittance) and in Zone 4, no voltage was applied until the initial transmittance was attained. Table S5 shows the switching times of the ECDs using the three different electrolytes. The switching time is the time required to reach 90% of the transmittance when completely colored or bleached. Among the three electrolytes, the LiTFSI electrolyte had the slowest switching time in the open circuit, PSSA had the fastest switching time and when LiTFSI and MXene-PSSA were used together, the advantage of the fast-switching time of PSSA was obtained. Because Li^+^ ions are larger than H^+^ ions, the switching time is relatively long.

**Figure 7:**
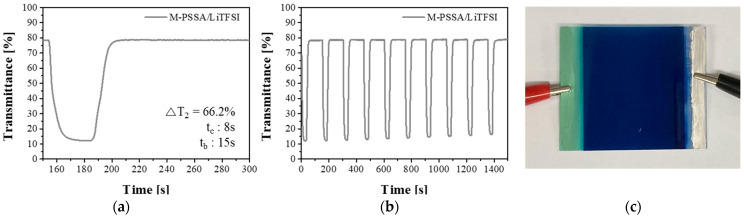
An optimized electrochromic device (ECD) and optical properties of the ECD. Applied square-wave potential between −1.2 V (30 s) and 0.5 V (120 s). (**a**,**b**) Transmittance vs. time of ECD, (**c**) A photograph of colored ECD (5 × 5 cm^2^).

When optimizing the ECD using the dual-cation electrolytes crosslinked with MXene, a very high transmittance contrast of 66.2% was obtained and excellent coloration and bleaching times of 8 s and 15 s, respectively, were obtained in a 5 × 5 cm^2^ device (Figure 7a–c).

## 4. Conclusions

In this study, by crosslinking MXene and PSSA, a synergistic effect of high ionic conductivity was obtained. Since MXene has a large number of -OH groups on its surface, it can combine with PSSA by causing a dehydration and condensation reaction. As a result, it creates a path for the conduction of positive ions and provides high ionic conductivity. In addition, when an electrolyte composed of a conventional acid or metal salt alone is applied to an ECD, a fast reaction rate and high CE cannot be obtained simultaneously. Therefore, an electrolyte based on a Dual-Cation was designed for high-performance ECDs. Dual-Cation electrolytes crosslinked with MXene were applied to an ITO-free, all-solution-processable ECD. The ECD showed a fast response time (8 s/15 s), high CE (340.6 cm^2^/C) and a transmittance contrast (66.7%).

## Data Availability

Data sharing not applicable.

## Supplementary Materials

The following are available online at https://www.mdpi.com/article/10.3390/nano11040874/s1. Figure S1. Capacitance of electrolytes according to the quantity of MXene (a) Potential vs. time of ECD when using PSSA-based electrolytes (b) Potential vs. time of ECD when 0.25 wt% of MXene was added to the mass of PSSA (c) Potential vs. time of ECD when 0.5 wt% of MXene was added to the mass of PSSA (d) Potential vs. time of ECD when 1 wt% of MXene was added to the mass of PSSA. Figure S2. Transmittance vs time of ECDs of various types of electrolytes when electrode is used as ITO (a) LiTFSI, (b) PSSA (c) 1:20 LiTFSI in PSSA. Figure S3. Summary of some electrochromic properties in various electrolytes (a) MXene/Bis (trifluoromethylsulfonyl) amine lithium salt (MXene/LiTFSI) (b) poly(4-styrenesulfonic acid) crosslinked with MXene (M-PSSA) and (c) 1:20 LiTFSI in MXene-PSSA(M-PSSA/LiTFSI). Table S1. Ionic conductivity of electrolytes according to the quantity of MXene. Table S2. Capacitance of electrolytes according to the quantity of MXene. Table S3. Performances of ECDs based on MXene/LiTFSI electrolytes according to the quantity of LiTFSI compared to mass of acrylamide. Table S4. Response time of ECDs based on M-PSSA/LiTFSI electrolytes according to the quantity of LiTFSI compared to M-PSSA. Table S5. Summary of some electrochromic properties in various electrolytes.
