# Supplementary material for: Dual-Cation Electrolytes Crosslinked with MXene for High-Performance Electrochromic Devices
Source: Nanomaterials (Basel). 2021 Mar 30;11(4):874. doi: 10.3390/nano11040874 (PMC8065717; doi:10.3390/nano11040874)
Supplement: Supplementary file 1 [file nanomaterials-11-00874-s001.pdf]

nanomaterials

Supplementary Materials

Dual-Cation Electrolytes Crosslinked with MXene for High-Performance Electrochromic Devices

Soyoung Bae [1], Youngno Kim [2], Jeong Min Kim [1] and Jung Hyun Kim [1,*]

[1] Department of Chemical and Biomolecular Engineering, Yonsei University, 50 Yonsei-ro, Seodaemoon-Gu, Seoul 03722, Korea; sybae0922@yonsei.ac.kr (S.B.); jminjmin@yonsei.ac.kr (J.M.K.)
[2] KIURI Institute, Yonsei University, 50 Yonsei-ro, Seodaemoon-Gu, Seoul 03722, Korea; dudsh3@naver.com
* Correspondence: jayhkim@yonsei.ac.kr

Table S1. Ionic conductivity of electrolytes according to the quantity of MXene.

Sample	Ionic conductivity (S cm^{-1})
PSSA	2.8×10^{-3}
MXene 0.25wt%	6.3×10^{-3}
MXene 0.5wt%	3.5×10^{-3}
MXene 1wt%	2.6×10^{-3}

$$\sigma \text{ (Ionic conductivity)} = \frac{l}{RA}$$

l: Thickness of electrolytes (cm)

R: Bulk resistance of electrolytes (Ω)

A: Area of electrolytes (cm²)

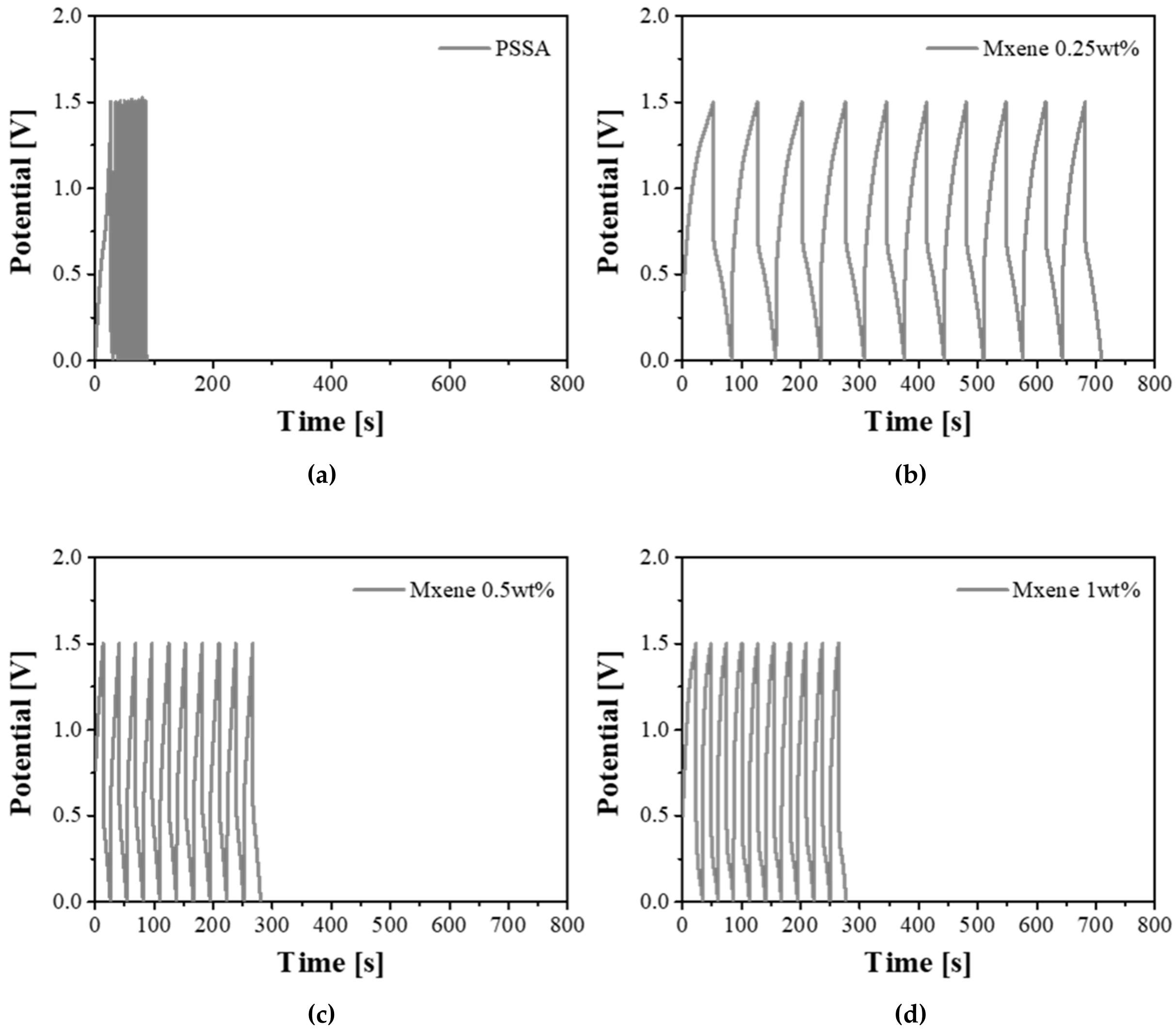

Figure S1. Capacitance of electrolytes according to the quantity of MXene (**a**) Potential vs. time of ECD when using PSSA-based electrolytes (**b**) Potential vs. time of ECD when 0.25 wt% of MXene was added to the mass of PSSA (**c**) Potential vs. time of ECD when 0.5 wt% of MXene was added to the mass of PSSA (**d**) Potential vs. time of ECD when 1 wt% of MXene was added to the mass of PSSA.

Table S2. Capacitance of electrolytes according to the quantity of MXene.

Sample	Capacitance (mF cm^{-2})
PSSA	0.16
MXene 0.25wt%	1.40
MXene 0.5wt%	0.59
MXene 1wt%	0.35

$$C\ (capacitance) = \frac{I\Delta t}{S\Delta V}$$

I: Discharging current (A)

$\triangle t$: Discharging time (s)

S: Surface area of the active area (cm^2)

$\triangle V$: Scanned potential window (V)

Table S3. Performances of ECDs based on MXene/LiTFSI electrolytes according to the quantity of LiTFSI compared to mass of acrylamide.

Sample	△T (%)	Coloration Time (s)	Bleaching Time (s)
LiTFSI 5wt%	50.6	9	600
LiTFSI 10wt%	57.4	10	75
LiTFSI 15wt%	56.7	12	283
LiTFSI 20wt%	57.4	12	1165

Table S4. Response time of ECDs based on M-PSSA/LiTFSI electrolytes according to the quantity of LiTFSI compared to M-PSSA.

LiTFSI : M-PSSA	△T (%)	Coloration time (s)	Bleaching time (s)
1:30	51.6	3	12
1:20	58.4	8	9
1:10	58.1	14	24

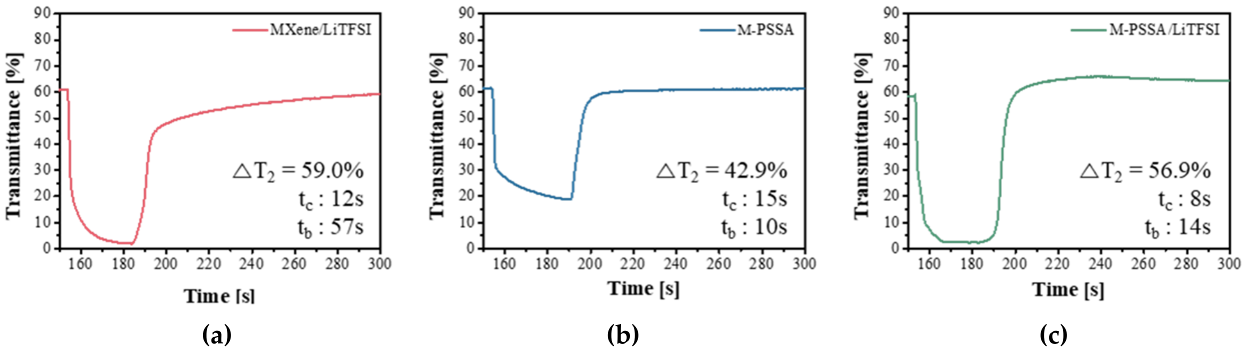

Figure S2. Transmittance vs time of ECDs of various types of electrolytes when electrode is used as ITO (**a**) LiTFSI, (**b**) PSSA (**c**) 1:20 LiTFSI in PSSA.

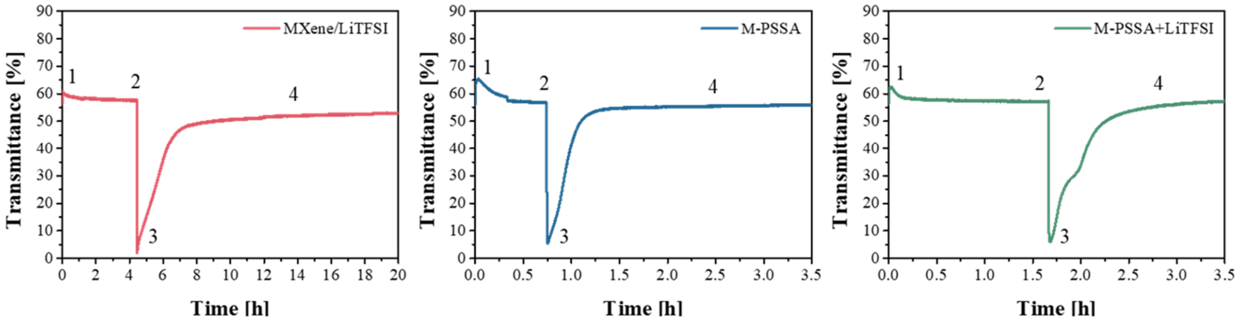

(a) **(b)** **(c)**

Figure S3. Summary of some electrochromic properties in various electrolytes (**a**) MXene/Bis (trifluoromethylsulfonyl) amine lithium salt (MXene/LiTFSI) (**b**) poly(4-styrenesulfonic acid) crosslinked with MXene (M-PSSA) and (**c**) 1:20 LiTFSI in MXene-PSSA(M-PSSA/LiTFSI)

Table S5. Summary of some electrochromic properties in various electrolytes.

**Electrolyte**		**LiTFSI**	**PSSA**	**1:20 in PSSA**
Switching time	1 → 2	4h 24m 8s	27m 55s	1h 4m 3s
	3 → 4	8h 38m 44s	23m 42s	41m 26s
